# Genome-Wide Identification and Characterization of the BRD Family in Nile Tilapia (*Oreochromis niloticus*)

**DOI:** 10.3390/ani12172266

**Published:** 2022-09-01

**Authors:** Chunmei Xu, Miao Yu, Qingqing Zhang, Zhisheng Ma, Kang Du, Huiqin You, Jing Wei, Deshou Wang, Wenjing Tao

**Affiliations:** Key Laboratory of Freshwater Fish Reproduction and Development (Ministry of Education), Key Laboratory of Aquatic Science of Chongqing, School of Life Sciences, Southwest University, Chongqing 400715, China

**Keywords:** BRD, evolution, expression profile, gonadal development, Nile tilapia

## Abstract

**Simple Summary:**

Nile tilapia is a good model for genome-wide identification and examination of the expression and role of gene families. In this study, we identified 54 bromodomain genes (BRDs) divided into eight subfamilies in Nile tilapia. Phylogenetic analysis revealed a high conservation of the BRDs family in vertebrates, with BRDs expansion due to fish-specific duplications. Most of the BRDs displayed sexually dimorphic expression in the gonads at 90 and 180 dah (days after hatching), including 21 testis-dominated genes (*brdt*, *brd4a* and *brd2b*, etc.), and 9 ovary-dominated genes (*brd3b*, *brd2a* and *kat2a*, etc.). Male fish treated with JQ1 (BET subfamily inhibitor) displayed abnormal spermatogenesis. The numbers of germ cells were reduced and the expression of steroidogenic enzyme genes was downregulated, while the expression of apoptosis-promoting genes was elevated in the testes of treated fish.

**Abstract:**

The bromodomain (BRD) proteins specifically recognize the N-acetyllysine motifs, which is a key event in the reading process of epigenetic marks. BRDs are evolutionarily highly conserved. Over recent years, BRDs attracted great interest because of their important roles in biological processes. However, the genome-wide identification of this family was not carried out in many animal groups, in particular, in teleosts. Moreover, the expression patterns were not reported for any of the members in this family, and the role of the BRD family was not extensively studied in fish reproduction. In this study, we identified 16 to 120 BRD genes in 24 representative species. BRDs expanded significantly in vertebrates. Phylogenetic analysis showed that the BRD family was divided into eight subfamilies (I–VIII). Transcriptome analysis showed that BRDs in Nile tilapia (*Oreochromis niloticus*) exhibited different expression patterns in different tissues, suggesting that these genes may play different roles in growth and development. Gonadal transcriptome analysis showed that most of the BRDs display sexually dimorphic expression in the gonads at 90 and 180 dah (days after hatching), including 21 testis-dominated genes (*brdt*, *brd4a* and *brd2b*, etc.), and nine ovary-dominated genes (*brd3b*, *brd2a* and *kat2a*, etc.). Consistent with transcriptomic data, the results of qRT-PCR and fluorescence in situ hybridization showed that *brdt* expression was higher in the testis than in the ovary, suggesting its critical role in the spermatogenesis of the tilapia. Male fish treated with JQ1 (BET subfamily inhibitor) displayed abnormal spermatogenesis. The numbers of germ cells were reduced, and the expression of steroidogenic enzyme genes was downregulated, while the expression of apoptosis-promoting genes was elevated in the testis tissue of treated fish. Our data provide insights into the evolution and expression of BRD genes, which is helpful for understanding their critical roles in sex differentiation and gonadal development in teleosts.

## 1. Introduction

Bromodomain (BRD) proteins include a protein domain with approximately 110 amino acids [1,2], comprising four α-helices (αZ, αA, αB, αC) linked by ZA and BC loops. Recognizing the acetylated lysine binding site [3,4], BRD proteins play different roles in biological processes through various mechanisms. For example, these proteins can act as a scaffold for larger protein complexes to facilitate their assembly, function as transcription factors and transcriptional co-regulators [5], and engage in various chromatin modifications [6,7]. Bromodomain was first identified in the fruit fly [8], and later in humans [3,5] and the buffalo [9]. However, to date, no study was systematically performed to analyze the evolution of the BRD family.

BRDs are ubiquitously expressed in the brain, testis, ovary, liver, spleen, lung, kidney and other tissues, with varying expression levels. For example, *brd2* is expressed in the zebrafish egg, early embryo, and nervous system [10]. *Brdt* is specifically expressed in murine testis [11]. *Brd1* is widely expressed in the mammalian brain and plays several roles in brain development [12]. *Brd7* is expressed in human ovarian cells and involved in epithelial ovarian carcinoma [13]. *Brd8* is expressed in the lung and coordinates the transcriptional network in human airway epithelial cells [14]. Despite its functional importance, the existing studies mainly focused on specific members of BRDs in humans, mice, and zebrafish. The expression level of all genes in this family and in other vertebrates, especially in non-model teleosts, was not yet studied. Advances in transcriptome sequencing make it possible to study the expression of all BRDs in different tissues at different developmental stages in vertebrates.

The BET subfamily is characterized by an N-terminal dibromodomain [15] and a unique C-terminal extra terminal (ET) domain [16], comprising Brd2, Brd3, Brd4, and the testis-specific protein, Brdt. This subfamily plays a key role in regulating gene transcription through the interaction between bromodomains and acetylated histones in cell multiplication and differentiation [17,18]. The BET subfamily is required for proper spermatogenesis in vertebrates. BET subfamily members were reported to be commonly expressed in the Sertoli cells of mouse testes [19] and involved in the regulation of testis development and spermatogenesis [20,21,22]. BET subfamily members are highly expressed in mature buffalo Sertoli cells, indicating their critical roles in spermatogenesis [19]. In gilthead seabream and European seabass, *brdt* had higher expression in gonads and relatively weak expression in other tissues [23]. Moreover, inhibition of *brdt* can induce contraception in male mice [22]. Disruption of *brdt* led to infertility in male mice [24]. A genome-wide association study indicated that single nucleotide polymorphisms of *brdt* were associated with oligospermia or azoospermia in humans [25]. However, to date, these studies were limited to the expression and function of individual genes or several members of the BET subfamily. Recently, treatment of BET inhibitor JQ1 (a thieno-triazolo-1,4-diazapine), which competitively binds to the acetyllysine recognition sites of BET family proteins, resulted in decreased numbers of spermatid cells and changes in chromatin accessibility in the mouse [26]. In vivo studies indicated that administration of JQ1 in male mice can lead to defective spermatogenesis and lower motility of spermatozoa [22]. However, the role of the BET subfamily was not extensively studied in fish reproduction.

Nile tilapia is an important aquaculture fish in the world, with the growth rate of males being faster than that of females. Because of the key role of BRDs in regulating gene expression, it is important to identify BRDs and characterize their expression patterns in different tissues in tilapia. The publication of the whole genome sequence, transcriptomes of eight tissues [27], and transcriptomes of gonads at four developmental stages [28], make it a good model for the genome-wide identification and examination of the expression and role of BRDs. In this study, we performed genome-wide identification of the BRD family and analyzed expression patterns. We injected, intraperitoneally, male tilapia with JQ1 to explore the possible function of the BET subfamily in male reproduction. Our study provides a new perspective on the evolution of BRDs and lays a foundation for elucidating their roles during fish spermatogenesis.

## 2. Materials and Methods

### 2.1. Fish and Experimental Conditions

The Nile tilapia used in this experiment were cultivated in the Key Laboratory of Freshwater Fish Resources and Reproductive Development of the Ministry of Education (Beibei, Chongqing, China), Southwest University. Fish were raised in a water circulation system at 26 °C, with the natural photoperiod (usually 12 h). Animal experiments were authorized by the Committee of Laboratory Animal Experimentation at Southwest University, China (No. IACUC-20181015-12).

### 2.2. Identification of BRD Genes in Representative Animals

In order to identify BRD genes in animals, we examined the genomes of 24 representative animals, including Drosophila melanogaster (GCA_000001215.4), Ciona intestinalis (GCA_000224145.2), Branchiostoma belcheri (GCA_001625305.1), Petromyzon marinus (GCA_010993605.1), Callorhinchus milii (GCA_018977255.1), Latimeria chalumnae (GCA_000225785.1), Python bivittatus (GCA_000186305.2), Xenopus tropicalis (GCA_000004195.4), Gallus gallus (GCA_016699485.1), Mus musculus (GCA_000001635.9), Homo sapiens GCA_000001405.29), Lepisosteus oculatus (GCA_000242695.1), Takifugu rubripes (GCA_901000725.2), Micropterus salmoides (GCA_014851395.1), Oreochromis niloticus (GCA_001858045.3), Oryzias latipes (GCA_002234675.1), Poecilia Formosa (GCA_000485575.1), Xiphophorus maculatus (GCA_002775205.2), Ictalurus punctatus (GCA_001660625.2), Pangasianodon hypophthalmus (GCA_009078355.1), Tachysurus fulvidraco (GCA_022655615.1), Danio rerio (GCA_000002035.4), Astyanax mexicanus (GCA_023375975.1), and Cyprinus carpio (GCA_018340385.1). All BRDs were identified by reciprocal best-BLAST against a protein database of these species, using human BRD-protein sequences as the query sequences [29]. The accession numbers of all BRD family-member gene sequences used in this study are listed in Table S1.

**Figure 1:**
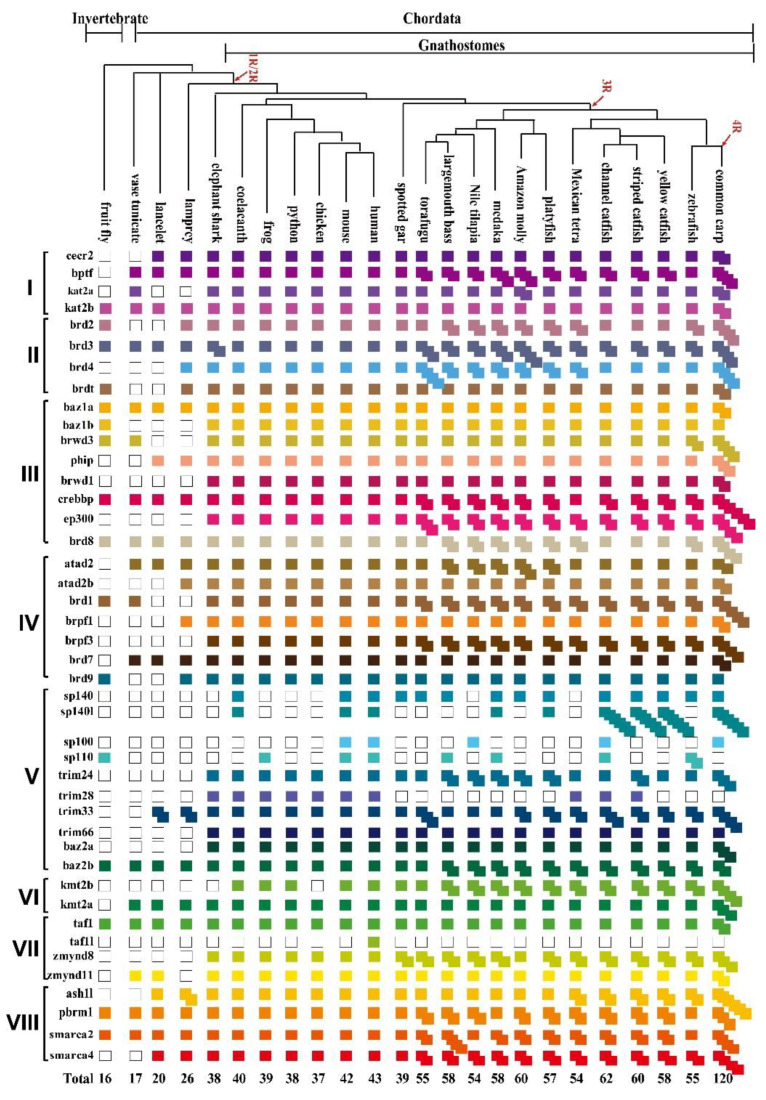
Phylogenetic relationship and numbers of BRD family members identified in 24 representative animals. The four rounds of whole genome duplication (WGD) are represented by 1R, 2R, 3R, and 4R, respectively. The fruit fly, *Drosophila melanogaster*; vase tunicate, *Ciona intestinalis*; lancelet, *Branchiostoma belcheri*; lamprey, *Petromyzon marinus*; elephant shark, *Callorhinchus milii*; coelacanth, *Latimeria chalumnae*; python, *Python bivittatus*; frog, *Xenopus tropicalis*; chicken, *Gallus gallus*; mouse, *Mus musculus*; human, *Homo sapiens*; spotted gar, *Lepisosteus oculatus*; torafugu, *Takifugu rubripes*; largemouth bass, *Micropterus salmoides*; Nile tilapia, *Oreochromis niloticus*; medaka, *Oryzias latipes*; Amazon molly, *Poecilia formosa*; Southern platyfish, *Xiphophorus maculatus*; Mexican tetra, *Astyanax mexicanus*; channel catfish, *Ictalurus punctatus*; striped catfish, *Pangasianodon hypophthalmus*; yellow catfish, *Tachysurus fulvidraco*; zebrafish, *Danio rerio*; and common carp, *Cyprinus carpio*.

The species tree shown in Figure 1 was constructed using the NCBI database Taxonomy Browser (https://www.ncbi.nlm.nih.gov/Taxonomy/CommonTree/wwwcmt.cgi (accessed on 5 October 2021)) [30], and then opened in Evolview (https://www.evolgenius.info//evolview/#login (accessed on 5 October 2021)) for editing.

### 2.3. Phylogenetic Analysis, Synteny Analysis, and Functional Domains Analysis of BRD Genes

The amino acid sequences of the BRD proteins of Nile tilapia, zebrafish and humans were aligned with Bioedit [31]. The phylogenetic tree was constructed by MEGA (version 7.0, Mega Limited, Auckland, New Zealand) using the neighbor-joining (NJ) method with a bootstrap of 1000 replicates to assess the confidence in the phylogeny [32], then edited with Adobe Illustrator (version 4.0, Adobe Systems Inc., San Jose, CA, USA). Syntenic analysis was conducted by comparing genomic regions that harbor *baz2b*, *crebbp*, *brd3*, *brd8*, *brd2*, *brd4*, *brpf3*, *ep300*, *brd,1* and *kmt2b* (representative genes underwent 3R-WGD) in tilapia with those in representative species. The genomic regions surrounding these genes were determined according to the Ensembl, NCBI, and Genomics databases [33] and edited with Adobe Illustrator CS4 software. NCBI Batch CD-Search (https://www.ncbi.nlm.nih.gov/Structure/cdd/wrpsb.cgi (accessed on 22 November 2021)) was used to search domains on BRD proteins. Gene structure was displayed by TBtools [34] and edited with Adobe Illustrator CS4 software.

### 2.4. Spatiotemporal Expression Pattern of BRDs in Tilapia

The transcriptomic data from eight tissues (ovary, testis, skeletal muscle, liver, kidney, head kidney, heart, and brain,) of adult tilapia were obtained from the NCBI database (Accession number: PRJNA78915 and SRR1916191) [27]. In our previous study, eight samples from the gonads of female (XX) and male (XY) fish at 5, 30, 90, and 180 dah (days after hatching) were sequenced using Illumina 2000 HiSeq technology [28]. A normalized measure of FPKM (fragments per kilobase million) was used to normalize the expression of BRD genes. BRDs with FPKM ≤ 1 in each sample were defined as background expression, according to a previous study [35]. The sexually dimorphic expression gene in the gonads was identified by fold-change ≥ 2 at cutoffs of *p* ≤ 0.01 using edgeR.

### 2.5. Validation of Spatiotemporal Expression Pattern of BRD Genes by qRT-PCR and Fluorescence In Situ Hybridization

The expression of *brdt* and *brd2b* was detected by quantitative real time PCR (qRT-PCR). In total, 12 tissues were isolated from Nile tilapia at 180 dah. Total RNA was extracted and a 1.0 µg total RNA template was reversely transcribed into cDNA following the method described previously [36]. The qRT-PCR was performed following the SYBR Green I Master Mix (Takara, Dalian, China) protocol. The relative abundance of mRNA transcripts was calculated by the formula R = $2^{-\Delta \Delta \mathrm{Ct}}$ [37]. *gapdh* was used as an internal control. Data (expressed as the mean ± SD) from all experiments were analyzed using GraphPad Prism 5.0. A one-way ANOVA was performed, followed by the Tukey test to determine the significance (* *p* < 0.05; ** *p* < 0.01; *** *p* < 0.001; ns, no significant changes observed).

Fluorescence in situ hybridization (FISH) was used to detect the expressed cell types of *brdt* in tilapia gonads at 180 dah. The cds of *brdt* was amplified with specific primers (Table S2), and the amplified fragments were ligated into pGEM-T Easy Vector. The gonads of male and female tilapia were sampled at 180 dah and fixed in 4% paraformaldehyde in phosphate-buffered saline (PBS), then embedded, and serial paraffin sections were cut at 5 µm thickness. The sections were dewaxed, rehydrated, and digested with proteinase K (4 µg/mL; Roche, Mannheim, Germany) for 15 min (37 °C), and then hybridized with DIG-labeled RNA probes for at least 16 h. After hybridization, the sections were washed with SSC for 10 min, DIG I (0.1 mol/L maleic acid, 0.15 mol/L NaCl, ph7.5) for 5 min, blocked with DIG II (1% bovine serum albumin in DIG I) for 30 min at room temperature, and incubated with Anti-DIG-POD (Roche, Mannheim, Germany) labeled with alkaline phosphatase at 1:2000 dilution for 30 min at room temperature. The sections were then washed with DIG1 buffer. After washing, the TSA Plus Fluorescein System (PerkinElmer, Boston, MA, USA) was used for the amplification of the hybridization signals. The nuclei were stained by DAPI (Invitrogen, Carlsbad, CA, USA) staining. Fluorescence signals were captured by confocal microscopy (Olympus FV3000) (Olympus, Tokyo, Japan). The probes and qPCR primer sequences [38,39] used in this study are listed in Table S2.

### 2.6. JQ1 Treatment

JQ1 was dissolved in DMSO. DMSO, PEG300, Tween-80, and saline were added in sequence at ratios of 5%, 40%, 5%, and 50%, respectively; the stock solution, which was diluted to 1.25 mg/mL, was prepared for freezing at −80 °C. Fish were treated intraperitoneally with 1.25 mg/mL JQ1 at 10, 20, and 30 dah, respectively. The control group was injected with the same dose of DMSO.

In order to investigate the role of BETs in gonadal development, the histology of tilapia testes treated with JQ1 and DMSO at 60, 90, and 120 dah was examined, respectively. Gonads were dissected from fish anesthetized with MS-222 (Sigma Aldrich, St. Louis, MO, USA) and fixed in Bouin solution for 24 h at room temperature. The fixed samples were processed as follows: gonads were dehydrated in 70%, 80%, 90%, and 95% ethanol for 15 min, and 100% ethanol, 3 times, for 5 min each time. They were then cleared in xylene and ethanol mixture (1:1) for 15 min, and continuously cleared twice in xylene for 30 min each time. Next, the gonads were placed in melted paraffin. Samples were sliced at a thickness of 5 µm using a Leica microtome (Leica Microsystems, Wetzlar, Germany). Finally, sections were stained with hematoxylin and eosin (H&E) [40]. The photos were taken under an Olympus BX51 optical microscope (Olympus, Tokyo, Japan). The qRT-PCR was performed to determine the expression levels of apoptosis-related genes, germ-cell marker genes and Sertoli-cell marker genes in JQ1-treated fish and control fish.

## 3. Results

### 3.1. Identification of BRDs from Tilapia and Representative Species

Given the fact that the whole genome duplication (WGD) can drive the expansion of gene families, we isolated BRD genes from the genome of 24 representative species by a homology-based search (Figure 1 and Table S1). The contig N50, scaffold N50, annotated gene numbers, and BUSCO information of 24 representative species are listed in Table S4. In the fruit fly, 16 BRDs were identified. In protozoa (vase tunicate and lancelet), 17–20 BRD genes were isolated. In jawless vertebrates (lamprey), 26 members were identified. In species that experienced the 2R WGD event, there was a significant expansion of the BRD family. In total, 38, 40, 39, 39, 38, and 37 BRDs were identified in the elephant shark, coelacanth, spotted gar, frog, python, and chicken, respectively. In total, 43 BRDs were isolated in humans, and all members in humans, except *taf1l,* were identified in the mouse. Further expansion of BRDs (54–62) was observed in teleosts experiencing the 3R event. Among those teleosts, 54 BRDs were isolated in tilapia. In common carp, a species that underwent the 4R event, 120 BRDs were isolated. Interestingly, *taf1l* was also lost during the evolution of teleosts. Furthermore, four speckled protein (SP) genes, *sp140*, *sp140l*, *sp110,* and *sp100,* were in mammals and channel catfish, but some were lost in other species. For example, *sp100* was lost in medaka, while *sp100* and *sp140l* were lost in zebrafish. All the four SP genes were absent in the Mexican tetra, python, and chicken. In addition, two to four copies of *brd2*, *brd3*, *brd4*, *baz2b*, *ep300*, *crebbp*, *brpf3*, *kmt2b*, *brd1,* and *brd8* were observed in teleosts, including largemouth bass, tilapia, medaka, Amazon molly, Southern platyfish and common carp, while only one copy was present in tetrapods, suggesting these genes were duplicated in the 3R-WGD. However, some of those genes were lost during the evolution of teleosts. For example, only one copy of *brd2* was found in torafugu, channel catfish, striped catfish, and yellow catfish.

### 3.2. Phylogenetic and Syntenic Analyses of BRD Members

**Figure 2:**
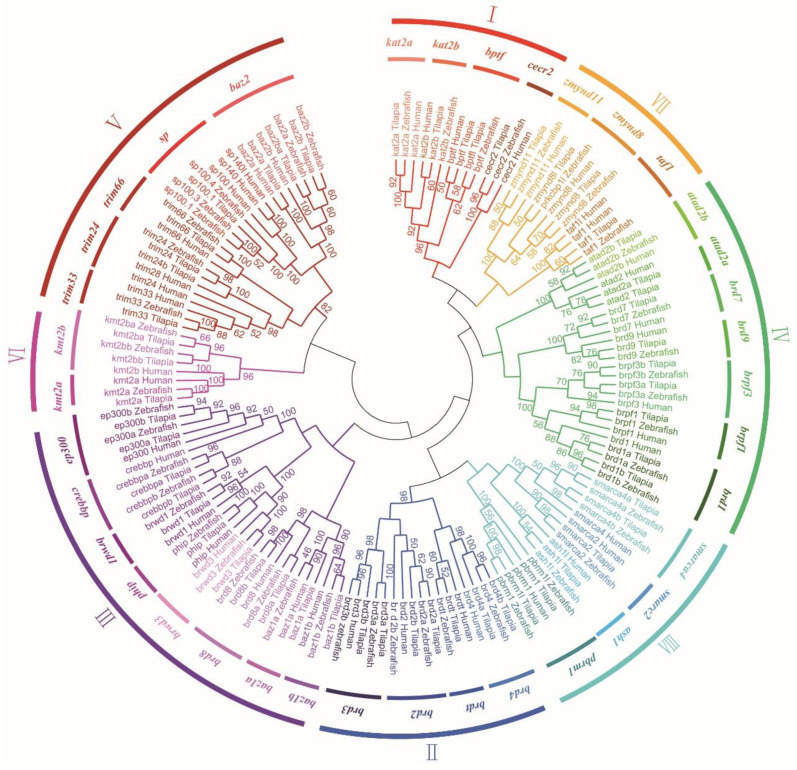
Phylogenetic tree of BRD family members of tilapia, humans and zebrafish. BRD family was divided into eight subfamilies (I–VIII). Numbers represent the bootstrap value. BRD protein sequences were aligned using Bioedit. The tree was constructed using NJ method in Mega 7.0. GenBank accession numbers are listed in Table S1.

Phylogenetic analysis was performed using the conserved domain of all BRDs from tilapia, humans, and zebrafish. The BRD family was divided into eight subfamilies (Figure 2). Subfamily I included four members (*cecr2*, *bptf*, *kat2a,* and *kat2b*). Subfamily II (the BET subfamily) included *brd2, brd3, brd4,* and *brdt*. Subfamily III included eight members (*baz1a*, *baz1b*, *brwd3*, *brwd1*, *phip*, *crebbp*, *ep300,* and *brd8)*. Subfamily IV comprised the ATPase proteins (*atad2* and *atad2b*), *brd1*, *brpf1*, *brpf3*, *brd7,* and *brd9*. Subfamily V included the speckled proteins (*sp100, sp110, sp140,* and *sp140l*), tripartite-motif-containing (TRIM) proteins (*trim24*, *trim28*, *trim33* and *trim66*), and *baz2*. Subfamily VI comprised *kmt2a* (*mll*) and *kmt2b*. Subfamily VII included four members (*zmynd8*, *zmynd8l*, *taf1,* and *zmynd11*). Finally, subfamily VIII included six members (*ash1l*, *pbrm1*, *pbrm1l*, *smarca2*, *smarca4,* and *smarca4l*). To understand the evolutionary history of BRDs after 3R, we performed syntenic analysis of *brd2*, *brd3*, *brd4*, *baz2b*, *ep300*, *crebbp*, *brpf3*, *kmt2b*, *brd,1* and *brd8* from representative tetrapods and teleosts (Figure S1). In most of the vertebrates investigated, the following genes, *tbr1*, *rbms1*, *acd302*, *pkp4*, *tanci*, *dap1*, and *wdsub1,* were found in the neighboring regions of *baz2b*, of which *tanci* existed in all tetrapods and duplicated in 3R species. Some genes, adjacent to *brd2*, *brd3*, *brd4*, *ep300*, *crebbp*, *brpf3,* and *kmt2b*, such as *tap1*, *wrdr5*, *akap8l*, *xpnpep3*, *slx4*, *mapk13,* and *igflr1*, existed with only one copy in the 3R species. Conserved synteny of *brd2*, *brd3*, *brd4*, *baz2b*, *ep300*, *crebbp*, *brpf3*, *kmt2b*, *brd1,* and *brd8*, and their upstream and downstream genes, was observed in tilapia, cave fish, zebrafish, and medaka. However, the neighboring region of these genes did not share conserved synteny with the fruit fly, vase tunicate or lancelet (data not shown).

### 3.3. Chromosomal Localization of BRD Genes and BRD Protein Domains in Tilapia

The 54 BRD genes in tilapia were unevenly distributed in its genome, covering 19 of the 22 linkage groups (LGs) (Figure S2). In total, seven BRDs were on LG4, and five BRDs were on LG6, LG20, and LG22, respectively. Only one BRD gene was on LG2, LG8, LG16, LG18, LG19, and LG23. Interestingly, paralogs of *brd4* and *crebbp* were found on LG4 and LG6, and paralogs of *brd2* and *kmt2b* were found on LG11 and LG22. This uneven distribution pattern was also found in zebrafish and humans (Table S5). To further characterize BRDs, we predicted the domain of these proteins in tilapia. As expected, all BRD proteins had at least one bromodomain (Figure S3). The BET subfamily members displayed two bromodomains. Members of subfamily Ⅵ had only one bromodomain, while Pbrml and Pbrm had five and six bromodomains, respectively. In addition to Kat2a, Kat2b, Cecr2, Brd8a, Brd8b, Brd7, and Brd9, BRD proteins had other domains besides the bromodomain, such as the ET domain, PHD domain, BAH, and HMG domain.

### 3.4. Expression and Distribution of BRDs in Eight Tilapia Tissues 

**Figure 3:**
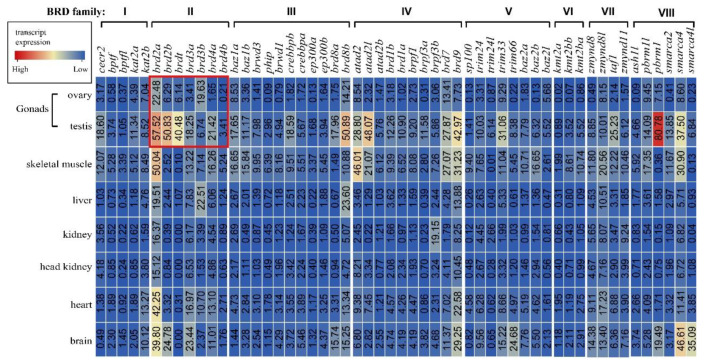
Transcriptome data of BRDs in eight tilapia tissues. Red shows high expression level and blue shows low expression level. Each column represents a different gene, and each row represents a different tissue.

Based on previously published transcriptomic data [28], the expression patterns of BRD genes in eight tissues from adult tilapia were analyzed, including ovary, testis, skeletal muscle, liver, kidney, head kidney, heart, and brain. Most BRD genes were expressed in at least one tissue (Figure 3). In total, 53 out of 54 genes were expressed in testis tissue, while *kmt2a* showed background expression level. Among these genes, the expression of *pbrm1* was the highest in testis tissue, while *brd2a* showed the highest expression in ovary tissue. Notably, *brd2a* was also the highest-expressed gene in muscle and heart tissues. *Brd8a*, *brpf3b,* and *smarca4* were the highest-expressed genes in liver, kidney and brain tissues, respectively.

### 3.5. Expression of BRDs in Tilapia Gonads at Different Stages

**Figure 4:**
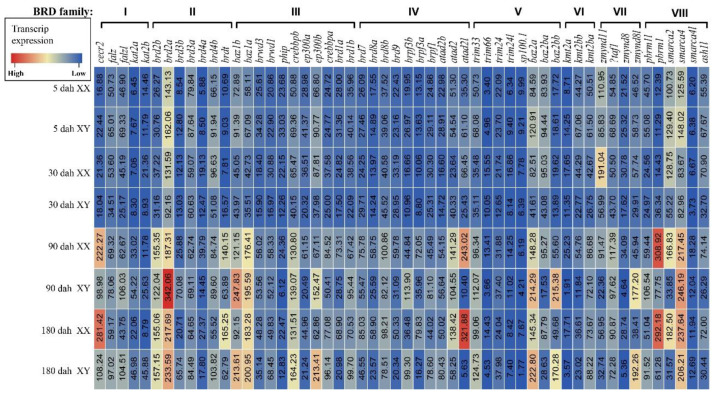
Transcriptomic data of BRDs at four developmental stages (5, 30, 90, and 180 dah) of Nile tilapia. A heatmap showing expression of BRDs in tilapia gonads at different developmental stages. Red shows high expression level and blue shows low expression level. dah, days after hatching.

The expression patterns of BRDs in XX and XY tilapia gonads at 5, 30, 90, and 180 dah (days after hatching) indicated all 54 BRD genes were expressed in at least one of the gonad samples (Figure 4). Interestingly, four genes (*zmynd11*, *sp100.1*, *phip,* and *brd3a*) were highly expressed and peaked at 5 dah (key stage of molecular sex determination), and then gradually decreased in later stages (30, 90, and 180 dah). The expression of other genes was at a relatively low level at early developmental stages (5 and 30 dah), with no significant differences between XX and XY gonads. Their expression gradually increased at 90 and 180 dah, and more than 55% (30/54) of those genes showed sexually dimorphic expression (Table S3) at 90 or 180 dah. Among them, 21 BRD genes (*cecr2*, *brd4a*, *brdt*, *phip*, *ep300a*, *brd8a*, *brpf3a*, *brd1a*, *atad2l*, *trim66*, *baz2b*, *kmt2a*, *kmt2bb*, *zmynd11*, *zmynd8*, *pbrm1*, *smarca2*, *ash1l*, *brd9*, *atad2,* and *sp100.1*) were highly expressed in testis tissue, while 9 BRD members (*bptfl*, *kat2a*, *kat2b*, *brd2a*, *baz1b*, *ep300b*, *brpf3b*, *baz2l* and *zmynd8l*) were dominantly expressed in ovary tissue. Interestingly, some paralogous genes displayed different expression patterns. For example, *ep300a* was highly expressed in testis tissue, while *ep300b* was highly expressed in ovary tissue.

### 3.6. Expression Validation and Cellular Location of BRDs

**Figure 5:**
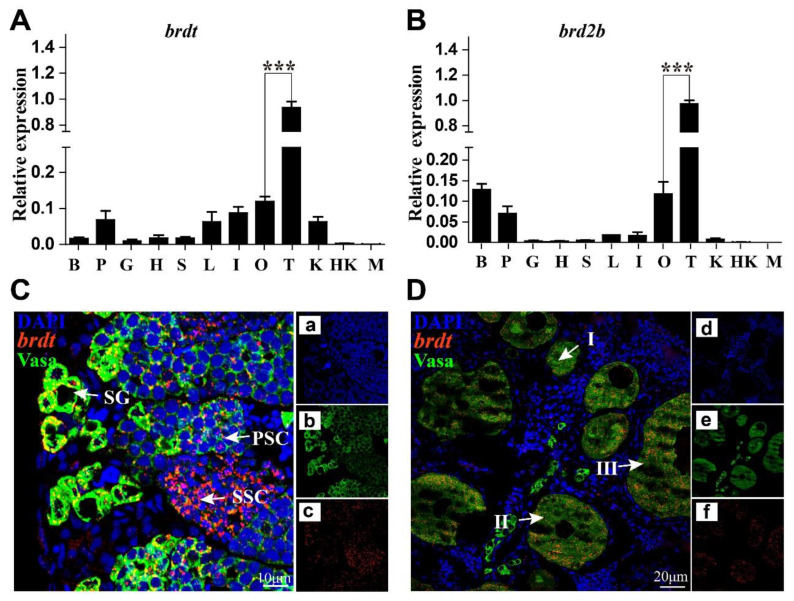
Expression validation and cellular location of BRDs. Expression validation of *brdt* and *brd2b* by quantitative real time PCR (**A**,**B**), and cellular location of *brdt* by fluorescence in situ hybridization in testis (**C**) and ovary (**D**) at 180 dah. Nuclei were counterstained with DAPI (a,d). Red fluorescence represents the signal of *brdt* (b,e). Green fluorescence represents the Vasa signals (c,f). *gapdh* was used as an internal control. Results are presented as mean ± SD. Asterisk above the error bar indicates statistical differences at *p* < 0.05 as determined by one-way ANOVA followed by Tukey test (*** *p* < 0.001). B, brain; P, pituitary; G, gill; H, heart; S, spleen; L, liver; I, intestinal; O, ovary; T, testis; K, kidney; HK, head kidney; M, muscle; I to III, phase I to III oocytes; SG, spermatogonia; PSC, primary spermatocytes; SSC, secondary spermatocytes. The white arrow indicates co-localization of Vasa and *brdt* signals.

To validate the transcriptome data, *brdt* and *brd2b* were selected for qRT-PCR validation. The expression of *brdt* and *brd2b* was consistently higher in testis tissue than in ovary tissue with qRT-PCR (Figure 5A,B). FISH was also performed for *brdt* to detect its cellular location in gonad tissue. Strong signals of *brdt* were observed in spermatogonia and spermatocytes of the testis, and signals were also detected in ovarian I, II and III oocytes (Figure 5C,D).

**Figure 6:**
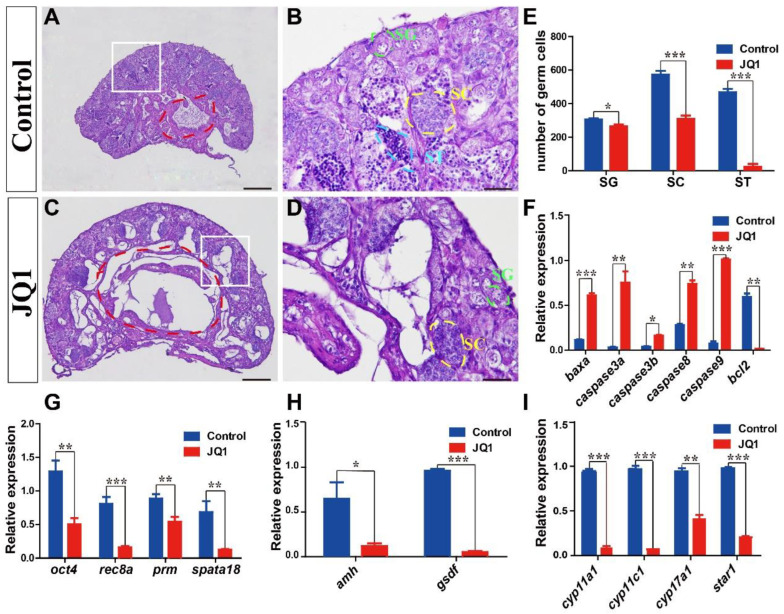
Inhibition of BET subfamily by JQ1 led to abnormal spermatogenesis. HE staining of Nile tilapia testis sections at 90 dah (**A**–**D**). The number of spermatogonia, spermatocytes, and spermatid in the testes of treated fish was significantly reduced, compared with that in the testes of control fish (**E**). The expression of apoptosis-related genes (**F**). Significantly decreased expression of germ-cell marker genes in the testes of treated fish, compared with that of control male fish (**G**). Significantly decreased expression of Sertoli-cell marker genes in the testes of treated fish, compared with that of control male fish (**H**). Significantly decreased expression of Leydig-cell marker genes in the testes of treated fish, compared with that of control male fish (**I**). *gapdh* was used as an internal control. Results are presented as mean ± SD. Asterisk above the error bar indicates statistical differences at *p* < 0.05 as determined by one-way ANOVA followed by Tukey test (*n* = 3–10; * *p* < 0.05, ** *p* < 0.01, *** *p* < 0.001). SG, spermatogonia; SC, spermatocytes; ST, spermatid. Scale bars 50 μm (A,C), 10 μm (B,D).

To explore the role of the BET subfamily in tilapia spermatogenesis, we injected Nile tilapia (10, 20, and 30 dah) intraperitoneally with the BET inhibitor JQ1. Morphological observations indicated that JQ1 treatment led to shrinkage of the seminiferous layer, with a reduced number of spermatogonia, spermatocytes, and spermatids, compared with the control fish (Figure 6A–E). The expression of the apoptosis-promoting genes *baxa*, *caspase3a*, *caspase3b*, *caspase8,* and *caspase9* was upregulated, while the expression of the apoptosis-inhibiting gene *bcl2* was downregulated in the testis of treated fish (Figure 6F). The qRT-PCR test revealed that the expression of germ cell-related genes *oct4* (spermatogonia), *rec8a* (spermatocytes), *prm* and *spata18* (spermatids) was significantly decreased in the testes of treated fish compared with that of control fish (Figure 6G). Compared with control fish, the expression of Sertoli-cell marker genes (*amh* and *gsdf*) in the testes of treated fish was significantly decreased (Figure 6H). Moreover, the expression of steroidogenic enzyme genes *cyp11a1*, *cyp11c1*, *cyp17a1,* and *star1* was also decreased in the testes of treated fish (Figure 6I). However, ovaries were not significantly different after JQ1 treatment compared to controls (Figure S4).

## 4. Discussion

BRDs, a family of evolutionarily conserved proteins, play a vital role in gene regulation by ordered recruitment, anchoring, and regulation of various chromatin-modifying factors. To date, identification and expression of BRDs were undertaken in only a few species. Previously, 43 different BRDs were identified and divided into eight subfamilies according to the sequence similarity in humans [3,5]. However, 22 BRDs were identified and classified into six groups on the basis of the conserved sequences in buffalo [9], which was incomplete and inconclusive. Therefore, the number and evolution of the BRD family require clarification. In this study, we identified BRDs in 24 representative animals using bioinformatic analysis. WGD usually generated duplicate homologous genes during evolution [41]. It is generally believed that three rounds of WGD occurred during vertebrate evolution. The first two rounds of duplication events (1R and 2R) occurred early in the vertebrate lineage, while the third duplication event (3R) occurred only in teleosts [42,43,44,45]. Some teleosts, such as common carp, have even undergone a fourth round of genome duplication (4R) [28]. This study indicates an expansion of the BRD family members via 2R, 3R, and 4R WGD. Although teleosts experienced the 3R-WGD [46], only 10 genes (*brd2*, *brd3*, *brd4*, *baz2b*, *ep300*, *crebbp*, *brpf3*, *kmt2b, brd1,* and *brd8*) had paralogs, and most duplicated genes were lost secondarily under selection pressure, as in the case of many other gene families [44,45]. The exact number of BRD genes might change slightly due to incomplete genome sequencing, assembly, and annotation. However, these will not change the expansion pattern of this family in jawed vertebrates due to whole genome duplication. Interestingly, the duplicated paralogs of *brd2* and *kmt2b* were on LG11 and LG22, respectively, similar to *cbx3*, *rps19,* and *rps27-1* [47,48], suggesting possible inter-chromosomal duplication in tilapia. Moreover, uneven chromosomal distribution of BRDs was observed in tilapia, zebrafish, and humans.

Bromodomains can recognize histone acetylation sites, and different numbers of bromodomains decide different binding affinity to histones among BRDs [49]. Thus, Pbrml and Pbrm might have a higher binding affinity because they contain five and six bromodomains, respectively. Moreover, the histone-binding sites of BRD proteins are selective. According to previous reports, Kat2a recognizes H3K27Ac [50], and Brd2 can bind H4K12ac [51] and H4K5acK12ac [52]. Brpf1 acetylates histones H2A, H2B, H3 and H4 [53], while Bptf binds specifically to H3K4me3 [54]. Pbrm1 interacts broadly with H3K36ac, H3K14ac, H3K115ac, H4K12ac, H2AK15ac, H2BK15ac, H3K122ac, H2BK24ac, and H2BK116ac [49]. Additional domains (e.g., ET, HMG, and PHD) of different BRDs contribute to specific histone-binding site recognition.

Gene expression analysis is essential for understanding protein functions in biological processes. Previous studies investigated the expression pattern of BRDs and indicated that these genes are widely expressed in different tissues at different stages. For example, *brd2*, *brd3,* and *brd4* are expressed in testis, ovary, brain, muscle and kidney tissue [3,55,56,57]. BRDs were consistently found to be ubiquitously expressed in different tilapia tissue based on transcriptomic data. Notably, these genes were not uniformly expressed in different tissues. *Brd8* was reported to play an important role in the development and progression of the human liver [58]. In the present study, *brd8b* was the highest expressed gene in the tilapia liver, indicating its possible role in liver development. *Brpf3* was shown to be critical in renal clear cell carcinoma (KIRC) [59]. Interestingly, *brpf3b* was the most highly expressed gene in the tilapia kidney, suggesting it may affect kidney development. *Smarca4*, a highly expressed gene in tilapia brain tissue, was shown to play an important role in the development of child brain stems [60,61]. *Zmynd8*, a gene preferentially expressed in the tilapia brain, plays an important role in *Xenopus* neural differentiation [62]. *Brd7*, highly expressed in the testes of tilapia, demonstrated its potential role in spermatogenesis and male fertility in the mouse [63]. Among the genes expressed in the tilapia ovary, *brd2a* exhibited the highest expression. A previous study showed that *brd2* was located in oocytes and involved in mitotic and possibly meiotic cell-cycle regulation in the mouse ovary [64]. Interestingly, different expression profiles of *brd2a* and *brd2b* paralogs were found in tilapia tissues, as is the case in zebrafish [10], supporting functional diversity of duplicated genes based on differences in structural domain configurations. Among the genes expressed in testis tissue, *pbrm1* showed the highest expression level, which was reported to play an important role in gonad primordium formation during embryogenesis of *C. elegans* [65]. However, no study was undertaken to clarify its function in fish testis tissue.

Most BRDs displayed sexual dimorphic expression during gonadal development in tilapia. In total, 21 BRD genes (*cecr2*, *brd4a*, *brdt*, *phip*, *ep300a*, *brd8a*, *brpf3a*, *brd1a*, *atad2l*, *trim66*, *baz2b*, *kmt2a*, *kmt2bb*, *zmynd11*, *zmynd8*, *pbrm1*, *smarca2*, *ash1l*, *brd9*, *atad2,* and *sp100.1*) were expressed higher in testes than in ovaries, suggesting they were potentially involved in testis development and the maintenance of spermatogenesis. For example, *cecr2* was highly expressed in adult spermatogonia, and disruption of *cecr2* led to compromised ability to fertilize oocytes in the male mouse [66]. *Ep300a* was also reported to be expressed higher in the testis tissue of the medaka and Mexican tetra [67]. On the contrary, nine BRD members (*bptfl*, *kat2a*, *kat2b*, *brd2a*, *baz1b*, *ep300b*, *brpf3b*, *baz2l* and *zmynd8l*) were dominantly expressed in ovarian tissue, suggesting their potential roles in ovary development. However, there is no study into the function of these genes in the ovary, which is worthy of further study. *Brdt* was reported to be testis specific [19] and later study found that it was also expressed at a relatively low level in mouse oocytes [68]. *Brdt* was proven to be essential for the normal progression of spermatogenesis [69], and loss of *brdt* produced an arrest of spermatogenesis [24,70], with severely impaired chromatin organization and absence of post-meiotic cells in mice [71]. In gilthead seabream, *brdt* was specifically expressed in secondary spermatocyte and spermatids, whereas in the ovary, *brdt* was expressed in late previtellogenic oocytes and early vitellogenic oocytes. In European seabass, *brdt* was expressed in late previtellogenic oocytes and spermatids [23]. In the present study, strong signals of *brdt* were observed in spermatogonia and spermatocytes of the testis, and moderate signals were also detected in I, II and III oocytes in tilapia. The cell location of *brdt* in tilapia testes was different from the previous studies in mouse, gilthead seabream, and European seabass, supporting the theory that there might be some interspecific differences in the functions of *brdt* [72].

The BET subfamily was reported to be involved in spermatogenesis [21]. In mice, *brd2* was expressed in spermatogonia, spermatocytes, and round spermatids [19]. *Brd3* was expressed in round spermatids, while *brd4* was expressed in spermatogonia [19]. Previous studies demonstrated that the BET bromodomain inhibitor JQ1 could penetrate the blood–testis barrier, and JQ1 treatment would result in reduced testis size, germ-cell defects, and reduced sperm motility and spermatid cells in mice [22,26]. In the present study, JQ1 treatment resulted in abnormal seminiferous layers and a reduced number of spermatogonia, spermatocytes, and spermatids, which closely resembles the findings in the characterization of *brdt* hypomorphic mice [24] and the potent inhibition of *brdt* in mice with JQ1 treatment [22]. However, we cannot rule out that JQ1 play its role through inhibition of other BET subfamily members which are also highly expressed in tilapia testes (e.g., *brd2b*, *brd4b* and *brd3a*). Previous studies showed that JQ1 could induce apoptosis [73], which was consistent with the upregulation of pro-apoptosis genes in JQ1-treated tilapia. Inhibition of *brdt* by JQ1 did not affect testosterone or gonadotropin levels in mice [22]. In the present study, the expression of *cyp11a1*, *cyp17a1,* and *cyp11c1* was downregulated in fish treated with JQ1, suggesting that the JQ1 treatment possibly blocked androgen synthesis in tilapia. However, the actual mechanism of JQ1 acting on gene expression in tilapia spermatogenesis remains to be elucidated. Because the BET subfamily may function as transcription factors, transcriptional co-regulators, and chromatin remodelers, we speculate that JQ1 treatment changed gene expression by altering chromatin conformation and, ultimately, led to abnormal spermatogenesis in tilapia, as in mice [26].

## 5. Conclusions

In this study, we completely identified the BRD family in 24 representative species by bioinformatic analyses. Differences in structures of the BRD members may be related to the diversity of their functions. Most BRD genes were found to be sexually dimorphic at different stages, with 21 genes being dominantly expressed in the testis and 9 genes dominantly expressed in the ovary, suggesting their important roles in gonad development. JQ1 treatment resulted in a reduced number of germ cells and abnormal spermatogenesis in tilapia. This study provided a new perspective on the evolution of the BRDs and laid a solid foundation for elucidating the role of the BRDs in vertebrates, especially in spermatogenesis.

## Data Availability

In this paper, all methods using fish material were carried out in accordance with relevant guidelines and regulations. The data used and/or analyzed during the current study were obtained from the NCBI website (https://www.ncbi.nlm.nih.gov/ (accessed on 5 October 2021)); the Ensembl Genomes database (https://ensemblgenomes.org/ (accessed on 22 November 2021)); and Genomicus (https://www.genomicus.bio.ens.psl.eu/genomicus-100.01/cgi-bin/search.pl (accessed on 22 November 2021)).

## Supplementary Materials

The following supporting information can be downloaded at: https://www.mdpi.com/article/10.3390/ani12172266/s1, Figure S1: Synteny of BRDs in representative species. Figure S2: Chromosomal maps of the BRDs in tilapia. Figure S3: Gene structure of BRDs in Nile tilapia. Figure S4: Morphological and histological analyses of the ovaries of treated fish. Table S1: The accession numbers of BRDs isolated. Table S2: Primers used in the present study. Table S3: The sex-dimorphic genes expressed in XX and XY gonads. Table S4: The contig N50, scaffold N50, annotated gene numbers, and BUSCO of the genomes of 24 representative species. Table S5: Chromosome distribution of BRDs in different species.
