# Supplementary material for: Genome-Wide Identification and Characterization of the BRD Family in Nile Tilapia (Oreochromis niloticus)
Source: Animals (Basel). 2022 Sep 1;12(17):2266. doi: 10.3390/ani12172266 (PMC9454494; doi:10.3390/ani12172266)
Supplement: Supplementary file 1 [file animals-12-02266-s001.zip › Figure S1-S4.pdf]

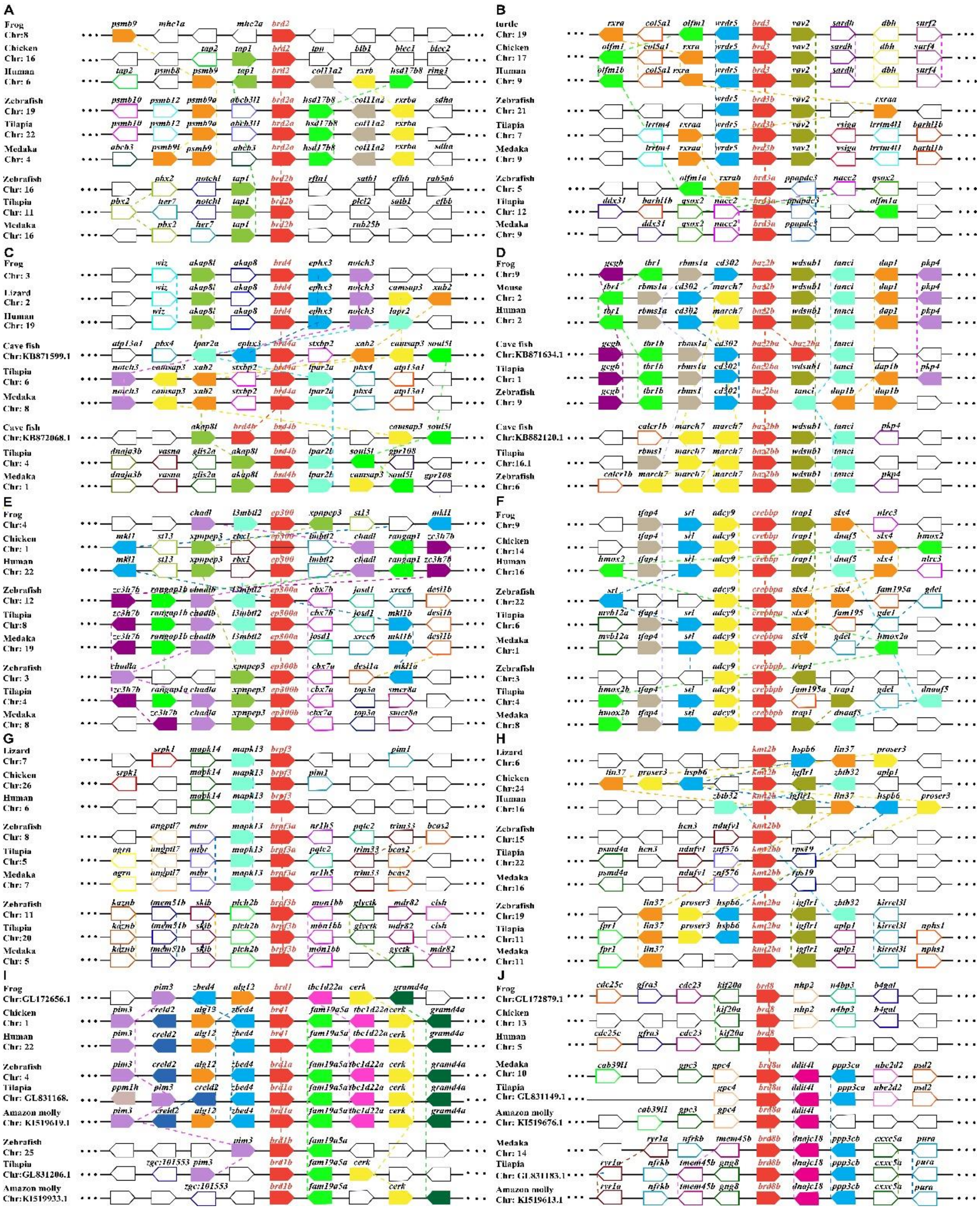

Figure S1. Synteny of BRDs in representative species. *brd2* (A), *brd3* (B), *brd4* (C), *baz2b* (D), *ep300* (E), *crebbp* (F), *brpf3* (G), *kmt2b* (H), *brd1*(I) and *brd8* (J). All syntenic genes were connected by dashed lines. Chromosomes numbers were shown at the beginning of each linkage group. Black dotted lines indicate genes omitted from chromosomes. The direction of the arrow indicates the gene orientation. Different colors indicate different genes, white arrows indicate those genes which have no collinearity in the drawn species.

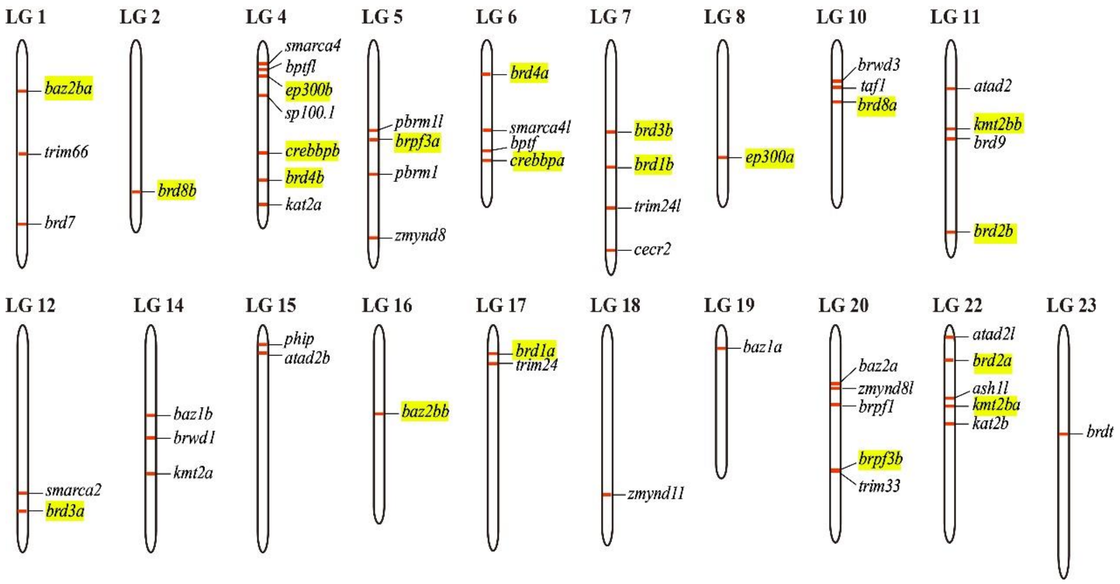

Figure S2. Chromosomal maps of the BRDs in tilapia. BRDs are depicted on a linkage group (LG) of the genome. BRDs were distributed on all chromosome except LG3, LG9 and LG13. LG21 is absent because it has been combined with LG16. The genes from the 3R event are highlighted in a yellow shade.

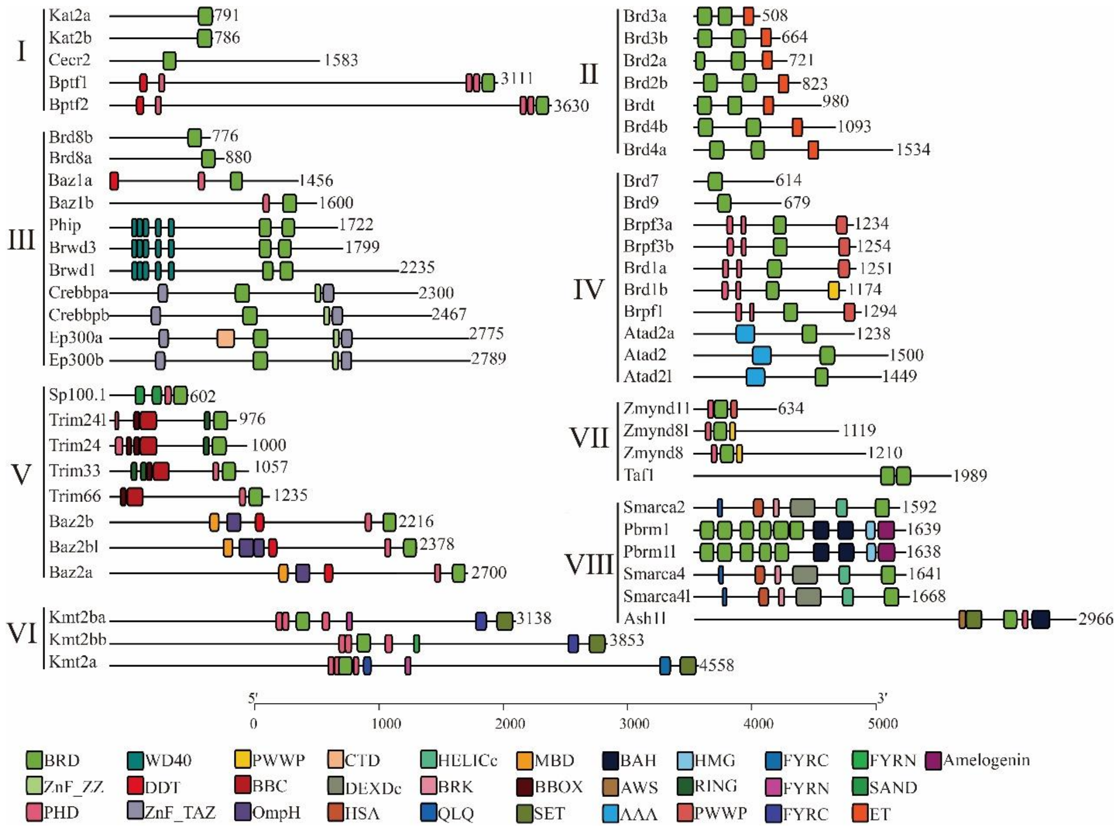

Figure S3. Gene structure of BRDs in Nile tilapia. Diagrams shown the conserved domains of BRDs predicted by the Batch CD-Search in NCBI, with the default E-value of 0.01. Rectangles in different colors indicate different domains, and the numbers indicate the length of proteins.

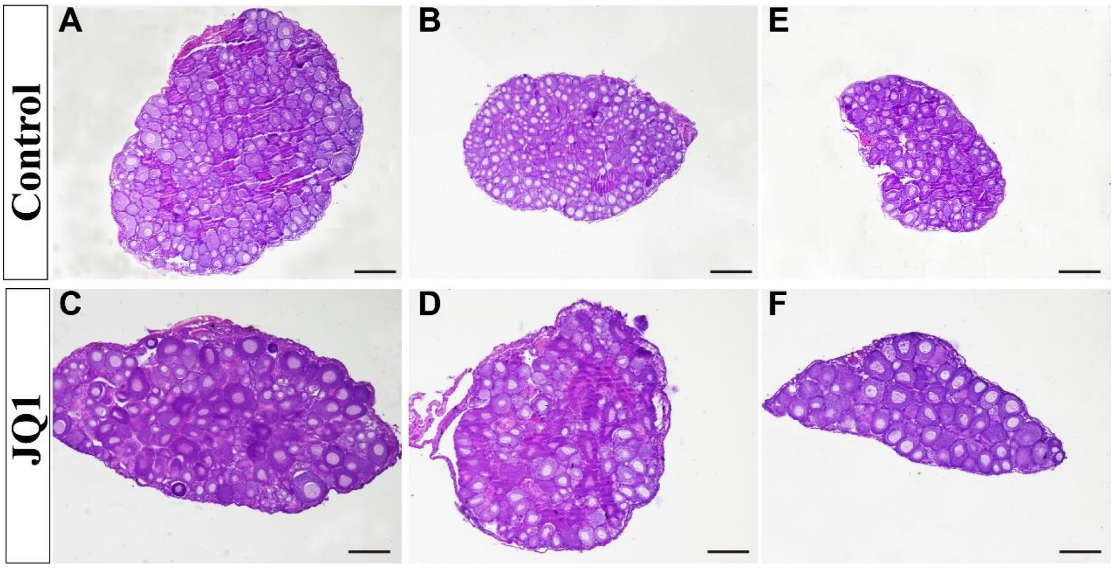

Figure S4. Morphological and histological analyses of the ovary of JQ1 treated fish. HE staining of JQ1-treated and control Nile tilapia ovary sections at 90 dah (A-F). No significant difference was observed between JQ1-treated and control fish. Scale bars 100 μm.
